# Educators’ digital competence in physiotherapy and health professions education: Insights from qualitative interviews

**DOI:** 10.1177/20552076241297044

**Published:** 2024-11-08

**Authors:** Yngve Røe, Astrid Cathrine Vik Torbjørnsen, Wilfried Admiraal

**Affiliations:** 1 Department of Rehabilitation Science and Health Technology, Faculty of Health Sciences, OsloMet—Oslo Metropolitan University, Oslo, Norway; 2 Department of Nursing and Health Promotion, Faculty of Health Sciences, OsloMet—Oslo Metropolitan University, Oslo, Norway; 3 Centre for the Study of Professions (SPS), OsloMet—Oslo Metropolitan University, Oslo, Norway

**Keywords:** Health professions education, educators, technology, digital competence

## Abstract

**Objective:**

This study seeks to outline the features of digital competences among educators in health professions education and pinpoint areas in need of enhancement.

**Methods:**

The transcribed interviews of nine educators in physiotherapy education were coded to align with The European Framework for the Digital Competence of Educators (DigCompEdu), adhering to a step-by-step procedure.

**Results:**

In total, 320 significant units were coded to an individual competence. Three competence areas (Professional engagement, Teaching and learning, and Empowering learners accounted for (94.2%) of the codes, while the three remaining (Digital resources, Assessment, and Facilitating learners’ digital competence) for 5.8% of cases. Several individual competences were not identified, across domains and the educators raised skepticism regarding the relevance of digital education for clinical practice.

**Conclusion:**

The study reveals deficiencies in the digital competence of health professions educators, highlighting gaps in strategies to utilize technology in their work and the integration of technologies with clinical skills. Educators exhibit individual-driven rather than collaborative digital professional development, expressing skepticism about technology’s efficacy in clinical skills training. The results emphasize the urgent need for comprehensive improvement. Without addressing these issues, health education students may graduate without essential digital skills, hindering their contribution to technology development.

## Introduction

Health professions education comprises study programs such as nursing, physiotherapy, occupational therapy, and pharmacy. Upon completing their studies, graduates in health professions education must possess the necessary knowledge and skills to fulfill their professional duties as health personnel efficiently, ethically, and safely. The digital transformation of society and health care changes work demands for health personnel and has fundamental implications for health professions education. To underscore a more future-oriented approach in Norwegian health education, a new governance system for the curricula of health professions and welfare education was introduced in 2019.^1^ This framework delineates 12 compulsory learning outcomes, with the final one specifying that candidates should demonstrate digital competence and the ability to contribute to the development and use of suitable technology at both individual and system levels upon graduation.^1^

In order to incorporate technologies effectively in line with governmental requirements, educators in higher education must also have the necessary skills. The Digital Education Action Plan (2021–2027) by the European Commission promotes digital competence as a vital skill for all educators and training personnel, to be weaved into every facet of their professional development.^2^ Digital competence is defined as the confident, critical, and responsible use and engagement with digital technologies for learning, work, and societal participation.^3^ The European initiatives in this realm were manifested in a recent digital action plan for Norwegian higher education, which suggests universities to take measures to enhance educators’ competence in the pedagogical use of digital technology to bolster student learning.^4^

In response to the rapidly changing digital landscape, the European Framework for the Digital Competence of Educators (DigCompEdu), was introduced in 2017.^5,6^ The DigCompEdu framework aims to delineate educator-specific digital competence by proposing 22 foundational abilities organized in the competence areas of (1) *Professional engagement*, (2) *Digital resources*, (3) *Teaching and learning*, (4) *Assessment*, (5) *Empowering learners*, and (6) *Facilitating learners’ digital competence*.^5^ Competence areas 2–5 encompass educators’ strategies to utilize technology in educators’ work, while area 1 addresses the broader professional environment, and area 6 details abilities required to facilitate students’ digital competence.^5^ The DigCompEdu provides a common language and a starting point for comparing and discussing digital competence among educators at all levels.

There is a rapidly growing body of literature on the digital competence of higher education educators and students, with three available systematic reviews.^7–9^ First, a comprehensive review that aimed to get an overview of the status and development of digital competence of students and teachers in higher education found that the majority of publications referenced EU policy and research to define digital competence.^9^ Moreover, a significant proportion of university students and teachers possessed only a basic level of digital competence.^9^ The authors noted that most studies evaluated educators’ digital competence by self-report, which may not show the real level of their digital competence.^9^ In terms of research methods, quantitative research methodology using questionnaires was applied by more than two-thirds of the studies.^9^

The second review aimed to analyze the research on digital competence of university educators, including the main thematic lines and the training needs of educators.^7^ The authors noted that in recent years there has been a notable and exponential increase in the number of publications on educators’ digital competence.^7^ A main finding was that across studies, educators’ digital competence fell below the medium level, particularly in competence areas related to the evaluation of educational practice.^7^ Based on these findings, the authors recommended that universities should develop practical and personalized training programs to strengthen the DigCompEdu.^7^

The third systematic review aimed at investigating the digital competence of higher education educators, the overall level was deemed moderate.^8^ A main finding was that Reflective practice in the *Professional engagement* area was not cited in any article, despite that this is often considered as a key competence for teachers and for improving current teaching.^8^ Also, the areas *Empowering learners* and *Facilitating students’ digital competence*, were little referenced in the studies.^10^ The authors of the review concluded that institutions should provide lifelong training to educators on digital competence.^8^

In addition to these reviews, an article, based on reanalyses of two previous reviews, focused on factors, which consistently, across borders and over time, influenced implementation of digital education in higher education study programs.^11^ Barriers such as educators’ teaching conceptions and their lack of digital competence have been cited as explanations for the reluctance of higher education to incorporate scholarly digital education.^11^ The article identified better alignment between research and teaching practices, a supporting infrastructure, and staffs’ professional development as prerequisites to succeed.^11^

Within health professions education, research on digital competence is limited. Findings from a quantitative survey among Finnish health professions educators suggest that certain areas of the DigCompEdu framework, specifically *Empowering learners* and *Facilitating learners’ digital competence*, may be perceived as weaker spots.^12^ Building on these findings, the authors strongly recommend institutional efforts to enhance educators’ digital competence within these areas.^12^ We are not aware of any other research that has assessed the digital competence of health professions educators within the reference of the DigCompEdu framework.

Health professions education must address the future competence demands for health personnel. A recent Norwegian governmental report recommended that, owing to demographic changes with an increasing number of elderly individuals, future health personnel will increasingly need to utilize digital technologies in their work.^13^ This shared reality, applicable to almost all Western countries, has sparked debate within the health professions disciplines. For instance, the World Confederation for Physical Therapy highlights the potential of modern technologies and digital practices to facilitate effective and impactful engagement with diverse audiences.^14^ Additionally, physiotherapy education has witnessed widespread use of pedagogical technology in the study program in the last decade, often showing promising outcomes in terms of students’ learning.^15^ However, concerns have also been raised that the pedagogical use of technology in physiotherapy education has primarily supported existing practices.^16^ Due to the strategic efforts within the physiotherapy community and the already considerable use of technology in physiotherapy education, it represents an interesting case for studying health professions educators’ digital competence.

This study aims to provide a deeper understanding of the DigCompEdu in physiotherapy, identifying strengths and areas for improvement. The research questions are as follows:

1. How can the key characteristics of digital competence among physiotherapy educators be effectively identified and described?
2. Which specific areas of digital competence among these educators require further development?

## Materials and methods

### Study design and data collection

The study employed a sequential, qualitative design with individual interviews followed by a focus group interview. The study adhered to the COREQ (COnsolidated criteria for REporting Qualitative research) Checklist (Supplementary file 1).^17^ The participants were a purposive sample of educators actively involved in teaching and the administration of digital teaching and learning in physiotherapy education. No subject exclusion criteria were applied in this study. For years, this particular study program had been active in pedagogical uses of digital technologies. In total, nine informants were recruited to the study, from January to March 2023. Of these, five were interviewed individually, while four took part in a focus group interview. According to gender, seven were women, and two were men. Eight of the educators had at least 5 years of teaching experience in the physiotherapy study program, while one educator had 2 years of experience. All participants had first-hand experience with digital education in teaching and/or leadership roles. The focus group interview was conducted using the conferencing platform Zoom (Zoom Video Communications, Inc.), while the individual interviews were conducted and recorded in person. The interview formats were chosen based on practical considerations. All interviews were conducted by the first author who had several years of experience as a researcher (×1). There was no direct relationship established with participants prior to study commencement.

First author (×1) verbatim transcribed the audio-recorded interviews. The transcripts in Word were used for textual analysis and sharing of notes as well as using NVivo for coding (https://lumivero.com/products/nvivo/).

Before conducting the interviews, a two-part interview guide was developed. The complete interview guide is provided as Supplementary file 2. The first part focused on four general themes:

- What are the opportunities and limitations of digital education in the physiotherapy study program?
- How does the pedagogical use of technology influence teaching and learning?
- How does the pedagogical use of technology influence the teaching role?
- Is the pedagogical use of technology in physiotherapy education merely a means to accomplish a purpose, or is it a purpose in itself?

To increase the dynamic in the conversation, preliminary themes generated from previous student interviews (not published) were used in the second part of the interviews. This method, named as stimulated recall, has been recommended to facilitate engagement among participants.^18^ The following themes had been generated from the student interviews:

- Digital education can impede social cohesion.
- Use of digital technology in education unhinged from professional practice.
- Digital education results in increased workload and facilitates the segmentation and distribution of tasks between students.
- No opinions about digital learning spaces.

### Analyses

The analysis spanned a 2-month period in autumn 2023 and was carried out by two researchers, ×1 and ×2. It was based on transcribed interviews and followed a stepwise process. Initially, the central meaningful unit of each account was determined. In the subsequent step, this unit was coded to the most relevant competence of the DigCompEdu framework. It might be quite easy to add the sentence about the most commonly used framework. Both of the researchers (×1 and ×2) had extensive experience with digital technology in health professions education. ×1 had first-hand experience with designing and implementing flipped classroom education in the physiotherapy study program. He also participated in the working group for the recent Norwegian digital education action plan.^4^ ×2 has worked with, and researched topics related to the pedagogical use of technology in nursing education and healthcare. Her research concerns how technology is being integrated into the curricula of health professions study programs.

Initially, both researchers independently coded three interviews and compared their results. Discrepancies and challenging cases were discussed until a consensus was reached. Building on this, ×1 recoded the same interviews and coded all remaining ones. Throughout this process, he noted cases that remained dubious. Based on shared written notes and oral discussions with ×2, ×1 finalized the coding. Both researchers agreed on the final version.

Throughout this stepwise coding process, several decisions were made regarding the interpretation of meaning and/or coding to the DigCompEdu framework. Examples of our coding decisions and the underpinning reasoning are presented in the table below and further elaborated in the Supplementary material.

In addition to the examples mentioned above, several key decisions had to be made during the analysis. Firstly, there were instances where informants did not explicitly reference digital technology in their pedagogical reflections. However, given that the interviews were conducted within the context of discussing digital education, we interpreted such instances as reflections on their digital educational practice. Secondly, in many cases, an informant’s comment had to be interpreted within the context of the topics under discussion since the text contained several implicit statements.

The DigCompEdu codes from the analysis were presented as frequencies (percentage) for the DigCompEdu competence areas and in nominal numbers for the specific abilities. To further display the opinions of the informants, a sample of frequent statements was reported.

### Ethical considerations

Each participant provided written informed consent prior to their involvement in the study. The study was approved by the Norwegian Centre for Research Data (ref. 178211), on 13 June 2022. The digital focus group interview was conducted according to the privacy policy for Zoom in research data storage at OsloMet—Oslo Metropolitan University. No copyrighted tools or materials were used in this study.

## Results

Altogether, 320 meaningful units were coded to a competence in the DigCompEdu framework. In addition, a single meaningful unit was decided as not covered by the framework.

### DigCompEdu competence areas

The distribution between competence areas is displayed in the below figure.

**Figure 1.**
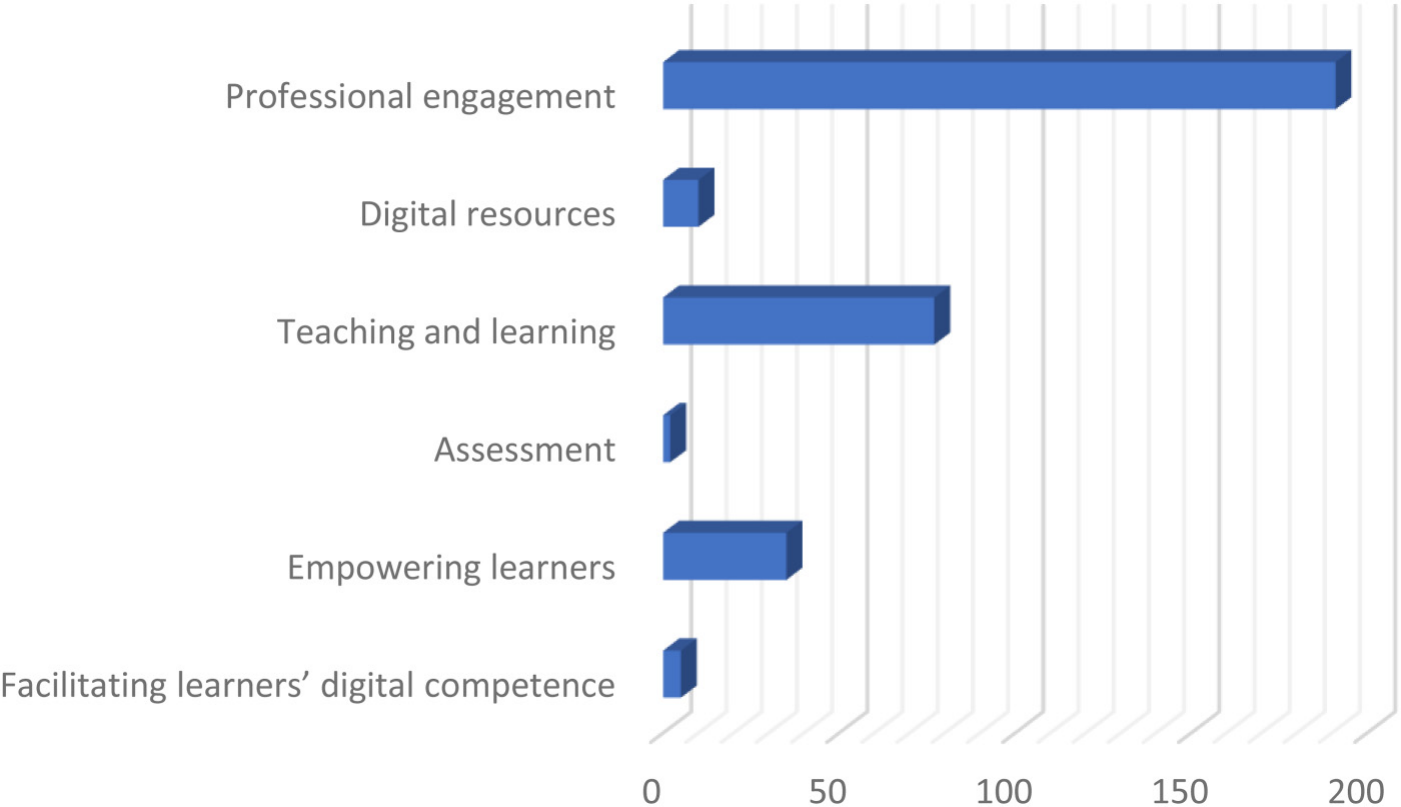
Distribution according to competence areas of the DigCompEdu framework.

As illustrated by Figure 1, three competence areas accounted for (94.2%) of the codes. Of these, *Professional engagement* accounted for 56% of the cases, *Teaching and learning* for 26.5%, and *Empowering learners* for 11.7%. The three remaining competence areas, *Digital resources*, *Assessment*, and *Facilitating learners’ digital competence*, accounted for 5.8% of cases.

### DigCompEdu competence areas and individual competences

The distribution of codes at the competence level in Table 1 shows the absence of codes within two of the lower frequent DigCompEdu competence areas. Within the competence area *Assessment*, the individual competences analyzing evidence and providing feedback and planning received no codes. Within the competence area *Facilitating learners’ digital competence*, the individual competences content creation, responsible use, and problem-solving received no codes.

**Table 1.** Examples of coding decisions during the analysis.

Narrative	Interpretation	Coding decision
“You should use digital media in teaching or learning, but students should also learn about digital … in other words, health technology and everything else that may not necessarily require digital teaching. They should also learn about the digital tools used out there. So, I think it's a bit like this, that we need to have both aspects in mind” (Informant 8).	Descriptions of their proficiency in utilizing digital technology and contemplations on its role in health professions education. Reflective practice encompasses pedagogical reflection within an educational context. Moreover, we opt to interpret this as reflective practice concerning digital solutions employed by the profession in practical settings.	*Professional engagement*, Reflective practice
“I believe that many will defend the learning methods they are used to using, and then they say that … and it is certainly true that many use some student-active learning methods, for example, case-based teaching and such. And it's not that I am negative towards that, but I have to say that I find the flipped [classroom] very interesting, because I think it is an opportunity to get people to prepare before teaching. And students say that when they come to the group and haven't prepared, it's very stigmatizing, and they notice that it's unusual. But colleagues … many are negative towards this” (Informant 1).	The essence of this statement lies in the occurrences within the classroom. The organizational communication category pertains to leveraging organizational communication channels to enhance interactions among various stakeholders in teaching. We have categorized this statement within this domain as it raises questions about how such organizational structures may pose challenges to the dynamics within the classroom.	*Professional engagement*, organizational communication
“Fortunately, we have quite a bit of freedom to choose the method when you are responsible for the subject. So, I think for me, there hasn't been resistance to the way I want to go, and it has been supported by my supervisor from day one as long as I have explained why and how. As for what colleagues would participate in, we have experienced that some choose not to be involved because they may need expertise in something they don't want to deal with at the moment” (Informant 2).	In the direct interpretation, the account delineates professional interactions with a supervisor and colleagues in the context of digital education. However, it is important to note that this comment was embedded within a more extensive conversation addressing the educators’ ability to repurpose digital resources. Consequently, we introduced a second code within the competence area of Digital Resources.	*Professional engagement*, Professional collaboration *Digital resources*, Managing protecting and sharing
“Yes, but I think maybe we could have broken it up a bit more. We could work on a part, then have some summaries, and then work on another part, with some summaries, so it doesn't become so monotonous” (Informant 3).	Referring to a targeted digital teaching strategy aimed at enhancing the teaching experience	*Teaching and learning*, Teaching
“The rooms now … many are set up for classroom teaching—it could be alright with some rooms adapted for work in smaller groups … a room with a screen in the middle, so that you can look at the same screen and know that there is a development that continues … and rooms that inspire skill training, not just a bench thrown in, but maybe slightly smaller rooms … rooms that stimulate creativity as a physiotherapist” (Informant 4).	Description of learning spaces as physical constraints for students’ digital learning. We opted to interpret this statement within the category of accessibility and inclusion. This category revolves around understanding how students engage with digital technologies, taking into account the physical environment where teaching occurs, and the number of individuals involved, as these factors can influence the inclusive nature of the learning experience for students.	*Empowering learners*, Accessibility and inclusion

As displayed in Table 2, there was a large variation in the distribution of individual competences within the three higher frequent competence areas. Relative to their numbers, some individual competences within these areas were highly underrepresented, these were: Within, Organizational communication and Professional collaboration (*Professional engagement*), Differentiation, and personalization (*Empowering learners*), and Self-regulated learning (*Teaching and learning*).

**Table 2.** Distribution of DigCompEdu codes at competence level.

Competence areas	Individual competences	Number of codes (*N* = 320)
Professional engagement	Organizational communication	16
	Professional collaboration	11
	Reflective practice	131
	Digital continuous professional development	33
Digital resources	Selecting	3
	Creating and modifying	6
	Managing, protecting, and sharing	1
Teaching and learning	Teaching	30
	Guidance	32
	Collaborative learning	13
	Self-regulated learning	2
Assessment	Assessment strategies	2
	Analyzing evidence	–
	Feedback and planning	–
Empowering learners.	Accessibility and inclusion	26
	Differentiation and personalization	1
	Actively engaging learners	8
Facilitating learners’ digital competence	Information and media literacy	1
	Communication	4
	Content creation	–
	Responsible use	–
	Problem solving	–

### Examples of statements from informants

The Reflective practice competence within *Professional engagement* alone accounted for more than two-thirds of the codes. Many reflections described how the core of clinical practice cannot be fully captured by digital technologies, as illustrated by the following account:

But I agree, we discussed at a seminar what we want the students to end up with…. I know that transferring to interdisciplinary work, working in teams, etc… but still, working as a physio, you work extremely alone … not everything has to be group or presentation … because we don't want them to just become good presenters in themselves—because that doesn't matter, it has to lead to something else. I'm sceptical that everything is being pulled in one direction just because it's a trend … we must remember the variation! (Citation 1)

This informant portrays physiotherapy practice as a deeply individual and almost lonely endeavor, contrasting the collaborative learning environment experienced in the physiotherapy study program. The self-regulated nature of the flipped classroom learning seems to create tensions, according to the regulatory role of the educators. There is skepticism about the simulation of clinical situations, considered unique, different, and real, emphasizing the need for continuous control over students’ skills in this arena:

…It takes a long time before you see how patients are in communication with them, can't test it out … trying role-playing in skills … doesn't respond well to it, it becomes a bit artificial. At least it's discovered first in the first practical period…. (Citation 2)

Following this line of thought, an informant describes the pedagogical use of technologies almost as a filter, disturbing the sight and moving away from basic clinical skills within the physiotherapy profession:

We are moving away from what we should be doing, which is dealing with people … it's okay not to view digital as a goal. After all, we are supposed to touch people, communicate, and examine, and for that, physical presence is necessary. (Citation 3)

Although many accounts focus on the limitations of digital technology, there are examples of *Professional engagement* reflections that highlight the opportunities offered by technology. According to this informant, technology can be used to provide healthcare regardless of geography:

…We need to think differently. I'm very mindful that Norway is a large country with vast distances. And for the patient's sake, they've travelled to a hospital hundreds of miles away—how practical is that? If you can achieve the same through digital media. I attended a technology conference a couple of years ago, and there were perspectives like … this was from a medical standpoint, but it's clear that if you, as a doctor, have to convey a difficult message that someone has a serious illness, that they might die. It might also be appropriate for the patient to be at home in their own living room with loved ones nearby. That could be just as good as coming to the hospital, maybe, and receiving the same message. So, these are the little things that I think, okay, there's a lot we need to think through and learn from. (Citation 4)

Another competence within *Professional engagement*, namely Digital continuous professional development, was frequently coded. Accounts coded to this competence often concerned topics such as time-spending, generation differences, and necessary areas for digital professional development:

Yes, I think it's very complex. This is about digital competence. In any place, there's always a range in digital competence and in learning the technology. Throughout the years I've been a leader, I've seen significant variations. And I think one must also accept that. If you're closer to retirement age, it might be okay if you're not…. So that's one aspect, there are variations in that, but perhaps the most important barrier is when I think about the pedagogical aspect. There's a lot of focus on the technical and on Zoom and things like that but thinking innovatively about the pedagogical side is the next step. (Citation 5)

Within the second most frequent competence area, *Teaching and learning*, the individual competences *Teaching and guidance* were far more frequent than collaborative learning and self-regulated learning. In both of these examples, the informants refer to particular experiences they remember back from the pandemic:

I mean, I think some of the feedback we received, for example, when everything was digital, like lectures or videos, was that they became unsure about how well or perhaps how deeply, or broadly, or what should be the focus of what was being said in such a digital lecture. It might be easier to address that in the moment when you're standing and giving a physical lecture, or it could also be digital but live-streamed, that you get this question saying we don't need to focus too much on this, or this is important. Unless you've made that very clear or set the agenda in the digital realm, as in our course, and they can see it when they want, and so on. So, I think there were many such questions that actually came up, and they were a bit uncertain…. Even though I, or maybe we thought that what we were saying they should achieve was very clear, they were still a bit uncertain about how much or how deeply they should go. So, it's perhaps that fine-tuning that is harder for them to do as well. (Citation 6)

I think that must be absolutely right. There isn't much tactile sensation through the screen. No, but I understand, for those who…. It's both the interaction and the relationship among themselves as students or in the group they work in that becomes more distant digitally, and also, it's the fact that they necessarily don't have that closeness to us teachers…. I was a bit surprised by, actually, because I thought they are more digitally raised, have experience with it…. Unfortunately, the pandemic has lasted quite a long time, but still, when we had some digital guidance towards the end related to assignments, or we had such question sessions, almost none of the students turn on the camera, and they have been in the same class for two years, right, worked in groups together, and we're talking about a class size of a maximum of 30 people. (Citation 7)

Within *Empowering learners*, accessibility and inclusion were frequent individual competences. As illustrated by the following comment, the informants had opinions on the physical learning environment and its usefulness in digital education:

Yes, I can understand that. Now we have a lot of group work, right … but it's not really facilitated for that … tables that you put together, so it doesn't quite fit … and not structured in a way that the rooms and learning methods are aligned. (Citation 8)

The competence *Actively engaging learners* in *Empowering learners*, was frequently coded. Some informant stories were influenced by their experiences working with flipped classroom teaching, in which the whole idea is that students prepare beforehand:

Yes, but I think it works really well. It can work really well. But what is absolutely crucial is getting the students to prepare in advance, and they should understand that when they receive a set of tasks to work on in seminars, it can't be answered in two lines…. (Citation 9)

## Discussion

Drawing on the European Framework for the DigCompEdu as a framework, this study aimed to delineate the characteristics of the digital competence among educators in health professions education and identify areas requiring improvement.^5^ As illustrated in Figure 1, educators’ digital competence was predominantly concentrated (94.2%) in three competence areas: *Professional engagement*, *Teaching and learning*, and *Empowering learners*. In contrast, the remaining three areas, *Digital resources*, *Assessment*, and *Facilitating learners’ digital competence*, were minimally represented. The disproportionate distribution across competence areas implies a deficiency in educators’ strategies to utilize technology, essential for fostering students’ digital competence.

The significant number of codes within the *Professional engagement* category in our study contrasts with the limited exploration of this topic in the existing scientific literature.^8^ Parallel to our findings, a study on Finnish health professions educators identified *Professional engagement* as their strongest area, while rating *Facilitating learners’ digital competence* and *Empowering learners* as their weakest.^12^ We attribute the high frequency of *Professional engagement* in our study to the extensive post-pandemic discussions surrounding the pedagogical use of technology in higher education.

Notably, as illustrated by the example statements, the educators’ opinions often focused on the limitations of technology to provide learning of “real” clinical skills. Already before the pandemic, low digital competence among educators and their beliefs about teaching has been identified as a barrier for pedagogical use of technology in higher education.^11^ The skepticism among the educators in our study also resonates with previous findings in physiotherapy education, which revealed that educators do not acknowledge any relationship between pedagogical use of technology in the study program and clinical skills required in the physiotherapy profession.^19,20^

We believe this skepticism also mirrors a contrasting perspective to technology expressed by some physiotherapists immediately after the pandemic. While digital remote therapy was seen as a potential complement to their regular physiotherapy practice, there was no intention to continue with it post-pandemic.^21^ These opinions among clinical physiotherapists and physiotherapy educators indicate that digital technologies are viewed, at best, as a supplementary tool to existing practices rather than a paradigm shift requiring substantial changes in their approaches. This apparent hesitation among clinical physiotherapists stands in stark contrast to predictions outlined in strategy documents regarding the future use of technology among physiotherapists and health professionals.^13,14^

In one aspect, our findings diverged from previous research; while *Empowering learners* received a high rank in our study, it was among the lowest in the Finnish study on health professions educators.^12^ We attribute this difference to the fact that educators in our study were prompted to provide insights on students’ opinions regarding digital learning spaces (the second part of the interview guide). Importantly, reflections within this domain often revolved around the design of learning spaces, with limited consideration given to the relationships between digital technological infrastructure and students’ learning. Consequently, we believe that there is a need to expand educators’ knowledge and skills within this area.

The near absence of the competence area *Assessment* aligns with prior findings indicating a lack of attention to this area in the literature.^7^ It is notable that in both this low-ranked competence area and another, *Facilitating learners’ digital competence*, several individual competences did not receive any codes (refer to Table 2). These include individual competences such as providing digital feedback and facilitating students’ digital content creation and problem-solving. In contrast, educators relatively frequently mention specific digital teaching and learning experiences. We infer from these gaps in educators’ competence that, at best, educators can participate in digital teaching or supervision but lack the necessary skills to independently initiate such educational approaches.

It is noteworthy to observe the relatively low representation of certain individual competences within high-frequency competence areas. Specifically, within *Professional engagement*, organizational work, and professional collaboration were less frequently addressed compared to educators’ reflections on digital education and their professional development. Despite the ambitious digital goals outlined in current governmental strategies, these findings suggest that such strategies may not have significantly influenced educators’ perspectives. Moreover, we posit that the results indicate current health professions study programs are not effectively bridging the pedagogical uses of technology on campus with clinical professional skills. Numerous accounts illustrated educators’ high skepticism regarding the benefits of students’ digital learning in relation to their clinical skills.

Another crucial finding is the overall limited representation within the *Digital resources* domain, especially in the individual competences of managing, protecting, and sharing skills. These skills are crucial for disseminating and reusing digital education across diverse settings. To fully exploit digital materials, an updated digital infrastructure is essential. Interestingly, in Norway, the Agency for Shared Services in Education and Research (Sikt) provides a secure database for disseminating and sharing digital resources across higher education institutions. Despite its easy access, our experience suggests that this digital infrastructure is underutilized and not well-known among educators. Considering that managing digital resources is a fundamental skill for integrating any digital educational approach, these findings are concerning and emphasize the need for universities to promote the existing digital infrastructure among their educators.

In this study, the DigCompEdu framework served as a valuable tool for guiding the topics covered in the interviews. A key strength of the framework is its ability to link digital competence to institutional capacity building, rather than viewing it solely as an individual endeavor. However, to present a comprehensive picture, it was essential to complement the coding with specific examples from the interviews. Notably, within the Professional Engagement competence area, the framework lacks guidance on capturing personal opinions. Moreover, we believe that the current version of DigCompEdu falls short in addressing rapid technological changes, including the transformative potential of Artificial Intelligence on educational practices and student experiences.^22^ For this reason, we anticipate the need to refine the framework soon.

The overarching implications of our findings emphasize the necessity to enhance educators’ digital competence across various domains. The importance of this subject is further underscored by recommendations from the European Commission, emphasizing digital competence as a fundamental skill for all educators, to be integrated into all aspects of their professional development.^2^ However, based on our findings, this effort should not solely focus on the professional engagement of individual educators; it should also involve organizational changes, including leadership. There is evidently a need to bridge digital competence and professional clinical skills, a task contingent on increased collaboration between health professions education institutions and healthcare organizations.

## Limitations

No other work aligning educators’ accounts similarly with the DigCompEdu framework has been identified. Drawing definitive conclusions about the interpretations of the findings is challenging due to the nature of the interviews. It could be argued that the codes represent educators’ digital interests rather than their skills. Nevertheless, given the range of topics provided by the interview guide, we believe the codes offer a fair representation of educators’ digital competence. We also acknowledge that the exclusive focus on informants from physiotherapy education makes absolute generalizations to health professions education challenging. However, considering that physiotherapy has been an early adopter of digital education, we deem it a reasonable assumption that the conditions regarding digital competence are similarly concerning in other health professions study programs.

Next, the number of participants in this study was limited. However, we believe that data saturation was reached after coding the interviews of nine educators, as no new significant themes or competence areas emerged during the final interviews. The consistent identification of key competence areas, along with the repetition of patterns across the data set, confirmed that further interviews were unlikely to yield additional insights into the digital abilities of health professions educators.

Lastly, due to practical reasons, the interview formats consisted of individual interviews and a focus group interview. Although the same interview guide was used in both formats, we cannot rule out that this influenced the breadth or depth of the data.

Given these considerations, the shortcomings identified in our study may present an idealized picture of health professions education overall.

## Conclusions

The study findings indicate deficiencies in the digital competence of health professions educators, both in terms of the covered competence areas and the connections between technologies and clinical skills. The educators’ narratives reveal that their digital professional development is more individually driven than being part of an organizational or collaborative effort. Despite their substantial experience with the pedagogical use of technology in the physiotherapy study program, they express significant skepticism about the efficacy of technology in training professional clinical skills.

Practically, the results suggest that educators lack the requisite strategies to utilize technology to design digital education or support students in their digital learning. The overarching implications underscore the urgent need to enhance educators’ digital competence across various domains. Without comprehensive and continuous efforts for improvement in this area, health education students may graduate without possessing digital competence, hindering their ability to contribute to the development and use of suitable technology at both individual and systemic levels.

There is a clear need for universities to adopt an organizational approach to addressing these issues. Additionally, the ongoing debate within health professions education about integrating technology into curricula is crucial. Norway has a modernized digital infrastructure in higher education, and physiotherapy programs have been early adopters of technology in their curricula. Future research should explore whether these findings are representative across health professions programs and in other countries.

## Supplemental Material

sj-pdf-1-dhj-10.1177_20552076241297044 - Supplemental material for Educators’ digital competence in physiotherapy and health professions education: Insights from qualitative interviews Supplemental material, sj-pdf-1-dhj-10.1177_20552076241297044 for Educators’ digital competence in physiotherapy and health professions education: Insights from qualitative interviews by Yngve Røe, Astrid Cathrine Vik Torbjørnsen and Wilfried Admiraal in DIGITAL HEALTH

sj-docx-2-dhj-10.1177_20552076241297044 - Supplemental material for Educators’ digital competence in physiotherapy and health professions education: Insights from qualitative interviews Supplemental material, sj-docx-2-dhj-10.1177_20552076241297044 for Educators’ digital competence in physiotherapy and health professions education: Insights from qualitative interviews by Yngve Røe, Astrid Cathrine Vik Torbjørnsen and Wilfried Admiraal in DIGITAL HEALTH
