# Supplementary material for: Educators’ digital competence in physiotherapy and health professions education: Insights from qualitative interviews
Source: Digit Health. 2024 Nov 8;10:20552076241297044. doi: 10.1177/20552076241297044 (PMC11544660; doi:10.1177/20552076241297044)
Supplement: sj-docx-2-dhj-10.1177_20552076241297044 - Supplemental material for Educators’ digital competence in physiotherapy and health professions education: Insights from qualitative interviews [file sj-docx-2-dhj-10.1177_20552076241297044.docx]

**Guide for teacher interviews**

**Part 1**

The following themes and questions will serve as a point of departure for the discussion:

T1: Opportunities and limitations for digital education in the physiotherapy study program

Q1: Do you consider yourselves to be technologically competent teachers?

T2: Pedagogical use of technology and its influence teaching and learning

Q1: What does pedagogical use of digital technology entail?

T3: Teaching role

Q1: How does the pedagogical use of technology influence the teaching role?

T: The implications of integrating pedagogical use of technology in education

Q1: What demands does technology place on the classroom environment?

Q2: How does technology change learning pedagogy?

Q3: What ethical dimensions are associated with digital teaching?

**Part 2**

Themes generated from preliminary analysis of student interviews:

- Digital education can impede social cohesion.
- Use of digital technology in education unhinged from professional practice.
- Digital education results in increased workload and facilitates the segmentation and distribution of tasks between students.
- No opinions about digital learning spaces.
